# Improving the Stationary Entanglement of a Laguerre–Gaussian Cavity Mode with a Rotating Mirror via Nonlinear Cross-Kerr Interactions and Parametric Interactions

**DOI:** 10.3390/nano14171389

**Published:** 2024-08-26

**Authors:** Guilin Lai, Sumei Huang, Li Deng, Aixi Chen

**Affiliations:** 1 Key Laboratory of Optical Field Manipulation of Zhejiang Province, Department of Physics, Zhejiang Sci-Tech University, Hangzhou 310018, China; 202230106302@mails.zstu.edu.cn (G.L.); sumei@zstu.edu.cn (S.H.); 2 School of Science, Zhejiang Sci-Tech University, Hangzhou 310018, China; lideng75@zstu.edu.cn

**Keywords:** Laguerre–Gaussian cavity optorotational system, nonlinear cross-Kerr interaction, degenerate optical parametric amplifier, steady-state entanglement, LG-cavity field, rotating end mirror

## Abstract

Quantum entanglement is essential in performing many quantum information tasks. Here, we theoretically investigate the stationary entanglement between a Laguerre–Gaussian (LG) cavity field and a rotating end mirror in an LG-cavity optorotational system with a nonlinear cross-Kerr (CK) interaction and a degenerate optical parametric amplifier (OPA). We calculate the logarithmic negativity of the system to quantify the stationary entanglement. We examine the influence of various system parameters such as the cavity detuning, the strength of the nonlinear CK interaction, the parametric gain and phase of the OPA, the power of the input Gaussian laser, the topological charge of the LG-cavity field, the mass of the rotating end mirror, and the ambient temperature on the stationary entanglement. Under the combined effect of the nonlinear CK interaction and the OPA, we find that the stationary entanglement can be substantially enhanced at lower Gaussian laser powers, smaller topological charges of the LG-cavity field, and larger masses of the rotating end mirror. We show that the combination of the nonlinear CK interaction and the OPA can make the stationary entanglement more robust against the ambient temperature.

## 1. Introduction

The experimental achievement of the laser cooling of mechanical modes [1,2] to the quantum ground state has laid a solid foundation for further realizing non-classical quantum effects and their applications. Over the past few decades, significant progress has been made in various aspects of cavity optomechanics [3]. Optomechanical systems have potential applications in optical information processing [3]. They have been proven to function as all-optical memory elements [4] and have been proposed as a new technology for single-photon detection [5]. Moreover, the effective preparation of various quantum states in optomechanical systems has been a subject of extensive research [3]. Entangled states, in particular, are valuable quantum resources [6]. Notably, optomechanical systems provide a robust platform where scientists can utilize optomechanical interactions to create entanglement between the cavity field and the macroscopic mechanical motion [7]; this enables various studies of quantum entanglement in macroscopic systems [8], thereby bridging the quantum–classical boundary [9]. The essence of these systems lies in the linear momentum exchange between photons and mechanical oscillators [7]. Additionally, the exchange of photon angular momentum can be utilized to design a Laguerre–Gaussian (LG) cavity optorotational system [10,11,12,13,14], in which a LG-cavityty mode is coupled to a rotating end mirror. The LG-cavityty mode has a well-defined orbital angular momentum equal to lℏ per photon, where *l* is the azimuthal mode index or the topological charge [15]. It has been demonstrated that the rotation of macroscopic mirrors can be cooled to near their quantum ground state [10]. Based on this, more quantum effects have been explored, such as the entanglement between LG-cavity fields and rotating mirrors [11,12] and rotational mirror–mirror entanglement [13,14].

Researchers have continuously striven to achieve stronger optomechanical coupling. One innovative approach [16] involves introducing a quantum two-level system (qubit) into optomechanical systems. Experiments [17] have demonstrated that using a quantum two-level system (qubit or artificial atom) can facilitate nonlinear cross-Kerr (CK) coupling between a mechanical resonator and a microwave cavity mode, enhancing the radiation–pressure coupling by six orders of magnitude. This enhancement allows the system to approach a strong coupling regime. Studies have shown that nonlinear CK interaction can improve the entanglement between a cavity field and a moving mirror [18,19]. In addition, our previous work has shown that nonlinear CK interaction can enhance the stationary entanglement between an LG-cavity mode and a rotating end mirror [20]. The other method is placing a degenerate optical parametric amplifier (OPA) into an optomechanical cavity to increase the optomechanical coupling, which is based on the fact that the squeezed light can be generated from a degenerate OPA [21]. It has been reported that a degenerate OPA in an optomechanical system can generate the multipartite optomechanical entanglement among the fundamental optical mode, the second harmonic optical mode, and the mechanical mode [22] and can enhance the stationary entanglement between two charged mechanical oscillators coupled through a Coulomb interaction [23].

In this paper, we investigate the stationary entanglement between an LG-cavity field and a rotating end mirror in an LG-cavity optorotational system with a nonlinear CK interaction and a degenerate OPA. Under the stability conditions of the system, we discuss the influence of the cavity detuning, the strength of the nonlinear CK interaction, the parametric gain and phase of the OPA, the power of the incident Gaussian laser, the topological charge of the LG-cavity field, the mass of the rotating end mirror, and the ambient temperature on the stationary entanglement. Compared to the results in our previous work [20], we find that the addition of the OPA into the optical cavity can substantially enhance the stationary entanglement at lower input powers, smaller topological charges of the LG-cavity field, and larger masses of the rotating end mirror and can make the entanglement more robust against the environmental noise. The entanglement measure, the approximations used, and the computational methods were first introduced by Bhattacharya et al. [11] and used in our previous work [20].

We have organized this paper as follows. In Section 2, we start with an introduction of the theoretical model, give the Hamiltonian of the whole system, derive the Langevin equations of the system, and obtain steady-state solutions. In Section 3, we obtain the linearized quantum Langevin equations and use logarithmic negativity to quantify the stationary entanglement between the LG-cavity field and the rotating end mirror. In Section 4, we discuss the effects of the cavity detuning, the strength of the nonlinear CK interaction, the gain and phase of the OPA, the incident Gaussian laser power, the topological charge of the LG-cavity field, the mass of the rotating end mirror, and the ambient temperature on the stationary optorotional entanglement. A concluding summary is given in Section 5.

## 2. Model

**Figure 1:**
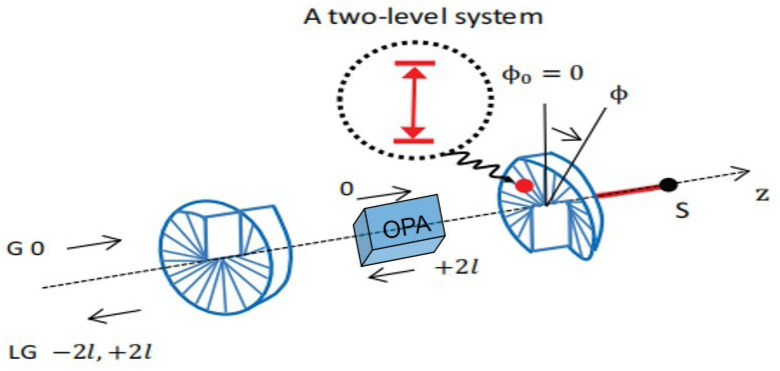
Schematic diagram of an LG-cavity optorotational system with a nonlinear CK interaction and a degenerate OPA. There is a two-level system (red) on the rotating end mirror to produce the nonlinear CK interaction between the LG-cavity field and the rotating end mirror. And a nonlinear optical crystal with a second-order nonlinearity is placed inside the cavity and is pumped by a laser to produce optical parametric amplification. The rotating end mirror is installed on the support S and can rotate about the *z* axis. The angular displacement of the rotating end mirror from its equilibrium position $\phi_{0}=0$ is denoted by ϕ. An external Gaussian laser beam (G) enters the optical cavity through the left fixed mirror. The value of the topological charge of each beam is shown.

The system we consider is an LG-cavity optorotational system formed by two cavity end mirrors with separation *L* [11], as depicted in Figure 1. The left fixed mirror transmits a small fraction of the incoming beam, and the right rotating mirror fully reflects the incoming beam. Both mirrors are spiral-phase plates [10]. The fixed mirror does not modify the topological charge of any laser beam transmitting through it and reduces a topological charge 2l from the laser beam during reflection. The rotating mirror adds a topological charge 2l to the laser beam during reflection. Thus, when a Gaussian laser beam is sent into the cavity, an LG-cavity field is built up inside the cavity. The LG-cavity field provides a torque $\frac{2l\hbar }{2L/c}=\hbar g_{\phi }$ to the rotating end mirror, where $g_{\phi }=\frac{cl}{L}$ is the optorotational coupling strength between the LG-cavity field and the rotating end mirror and *c* is the light speed in vacuum [10]. The rotating end mirror with mass *m*, frequency $\omega_{m}$, and damping rate $\gamma_{m}$ can be approximated as a harmonic oscillator. It is assumed that a quantum two-level system is introduced on the rotating end mirror, which generates the nonlinear CK interaction between the LG-cavity field and the rotating end mirror [17]. The strength of the nonlinear CK interaction is denoted by $g_{ck}$. The angular displacement operator ϕ and angular momentum operator $L_{z}$ of the rotating end mirror can be, respectively, written as $\phi =\frac{1}{\sqrt{2}}\left(b+b^{†}\right)$ and $L_{z}=\frac{1}{i\sqrt{2}}\left(b-b^{†}\right)$, where the annihilation and creation operators (*b*, $b^{†}$) of the rotating end mirror obey the commutation relation $[b,b^{†}]=1$. It is assumed that the frequency $\omega_{m}$ of the rotating end mirror is much lower than the cavity resonance frequency $\omega_{c}$. Thus, the dynamical Casimir effect can be ignored, which describes the generation of photons from the quantum vacuum due to the rotation of the end mirror [24]. Meanwhile, the rotational Doppler effect due to the angular momentum exchange during the optorotational interaction can also be neglected [25].

In a frame rotating at the incident Gaussian laser frequency $\omega_{l}$, the dynamics of the studied system is described by the following Hamiltonian:

(1) $$\begin{array}{ccc} H & = & \hbar \Delta_{0}a^{†}a+\hbar \omega_{m}b^{†}b-\hbar ga^{†}a\left(b+b^{†}\right)-\hbar g_{ck}a^{†}ab^{†}b+i\hbar G\left(e^{i\theta }a^{†2}-e^{-i\theta }a^{2}\right)\\ & \;\; & +i\hbar \varepsilon (a^{†}-a), \end{array}$$

where $\Delta_{0}=\omega_{c}-\omega_{l}$ is the detuning of the cavity field with respect to the input laser; *a* ($a^{†}$) is the boson annihilation (creation) operator of the LG-cavity mode; *g* is the optorotational interaction strength determined by $g=g_{\phi }\sqrt{\frac{\hbar }{2I\omega_{m}}}$, with $I=\frac{1}{2}mR^{2}$ being the moment of inertia of the rotating end mirror about the *z* axis and *R* being the radius of the rotating end mirror; *G* and θ are the parametric gain and phase of the OPA, respectively; and ε is the amplitude of the incident Gaussian beam determined by $\varepsilon =\sqrt{\frac{2\kappa ℘}{\hbar \omega_{l}}}$, with *℘* being the power of the incident Gaussian beam and κ being the cavity decay rate. In Equation (1), the fifth term represents the coupling between the LG-cavity field and the OPA. We assume that the degenerate OPA is driven by a pump field at frequency $2\omega_{l}$, and the three-wave mixing process in the OPA can result in the production of the signal and idler fields with identical frequencies $\omega_{l}$.

Using the Heisenberg equation of motion and considering the corresponding damping terms, we obtain the equations describing the motion of the optical and mechanical modes:

(2) $$\begin{array}{ccc} \dot{a} & = & -\left(\kappa +i\Delta_{0}\right)a+iga\left(b+b^{†}\right)+ig_{ck}ab^{†}b+2Ge^{i\theta }a^{†}+\varepsilon +\sqrt{2\kappa }a_{in},\\ \dot{b} & = & -\left(\frac{\gamma_{m}}{2}+i\omega_{m}\right)b+iga^{†}a+ig_{ck}a^{†}ab+\sqrt{\gamma_{m}}b_{in}, \end{array}$$

where we have included the input vacuum noise operator $a_{in}$ entering the cavity through the left end mirror and the thermal noise operator $b_{in}$ of the rotating end mirror associated with the Brownian motion of the rotating end mirror. Both the noise operators $a_{in}$ and $b_{in}$ have zero mean values. The correlation functions for the noise operators $a_{in}$ and $b_{in}$ in the time domain are given by

(3) $$\begin{array}{ccc} \langle a_{in}\left(t\right)a_{in}^{†}\left(t^{\prime }\right)\rangle & = & \delta (t-t^{\prime }),\\ \langle b_{in}^{†}\left(t\right)b_{in}\left(t^{\prime }\right)\rangle & = & n_{th}\delta \left(t-t^{\prime }\right),\\ \langle b_{in}\left(t\right)b_{in}^{†}\left(t^{\prime }\right)\rangle & = & \left(n_{th}+1\right)\delta \left(t-t^{\prime }\right), \end{array}$$

where $n_{th}=1/\left\{\exp\left[\hbar \omega_{m}/\left(k_{B}T\right)\right]-1\right\}$ is the thermal phonon number of the rotating end mirror, $k_{B}$ is the Boltzmann constant, and *T* is the temperature of the surrounding thermal environment. At the steady state, the mean values of the operators *a* and *b* are found to be

(4) $$\begin{array}{ccc} a_{s} & = & \frac{\kappa -i\Delta +2Ge^{i\theta }}{\kappa^{2}+\Delta^{2}-4G^{2}}\varepsilon ,\\ b_{s} & = & \frac{ig|a_{s}{|}^{2}}{\frac{\gamma_{m}}{2}+i{\bar{\omega }}_{m}}, \end{array}$$

where $\Delta =\Delta_{0}-g\left(b_{s}+b_{s}^{*}\right)-g_{ck}{\left|b_{s}\right|}^{2}$ and ${\bar{\omega }}_{m}=\omega_{m}-g_{ck}{\left|a_{s}\right|}^{2}$. $a_{s}$ and $b_{s}$ are the amplitudes of the LG-cavity field and the rotating end mirror at steady state.

## 3. Quantum Fluctuations

We assume that a strong Gaussian beam drives a single-mode LG-cavity field. In this case, the absolute amplitude $|a_{s}|$ ($|b_{s}|$) of the cavity (mechanical) mode is much larger than 1; thus, the two operators *a* and *b* can be replaced by $a=a_{s}+\delta a$ and $b=b_{s}+\delta b$, respectively, where δa and δb are small quantum fluctuations. We only keep the first order in the quantum fluctuations and obtain the following linearized equations for the fluctuations δa and δb:

(5) $$\begin{array}{ccc} \delta \dot{a} & = & -\left(\kappa +i\Delta \right)\delta a+ia_{s}g^{\prime *}\delta b+ia_{s}g^{\prime }\delta b^{†}+2Ge^{i\theta }\delta a^{†}+\sqrt{2\kappa }a_{in},\\ \delta \dot{b} & = & -\left(\frac{\gamma_{m}}{2}+i{\bar{\omega }}_{m}\right)\delta b+ia_{s}^{*}g^{\prime }\delta a+ia_{s}g^{\prime }\delta a^{†}+\sqrt{\gamma_{m}}b_{in}, \end{array}$$

where $g^{\prime }=g+g_{ck}b_{s}$.

For convenience, we introduce the quadrature fluctuations of the mechanical and cavity modes as $\delta \phi =\frac{1}{\sqrt{2}}\left(\delta b+\delta b^{†}\right)$, $\delta L_{z}=\frac{1}{i\sqrt{2}}\left(\delta b-\delta b^{†}\right)$, $\delta x=\frac{1}{\sqrt{2}}\left(\delta a+\delta a^{†}\right)$, and $\delta y=\frac{1}{i\sqrt{2}}\left(\delta a-\delta a^{†}\right)$, and the quadrature fluctuations of the input noises as $\phi_{in}=\frac{1}{\sqrt{2}}\left(b_{in}+b_{in}^{†}\right)$, $L_{zin}=\frac{1}{i\sqrt{2}}\left(b_{in}-b_{in}^{†}\right)$, $x_{in}=\frac{1}{\sqrt{2}}\left(a_{in}+a_{in}^{†}\right)$, and $y_{in}=\frac{1}{i\sqrt{2}}\left(a_{in}-a_{in}^{†}\right)$. The equations of motion for the quadrature fluctuations are given by

(6) $$\dot{U}\left(t\right)=AU\left(t\right)+N\left(t\right),$$

where U(t) represents the vector of the quadrature fluctuations of the mechanical and cavity modes, N(t) denotes the vector of the input noises, their transposes are

(7) $$\begin{array}{ccc} U{\left(t\right)}^{T} & = & (\delta \phi ,\delta L_{z},\delta x,\delta y),\\ N{\left(t\right)}^{T} & = & (\sqrt{\gamma_{m}}\phi_{in},\sqrt{\gamma_{m}}L_{zin},\sqrt{2\kappa }x_{in},\sqrt{2\kappa }y_{in}); \end{array}$$

and the 4×4 matrix *A* has the form

(8) $$A=\left(\begin{array}{cccc} -\frac{\gamma_{m}}{2} & {\bar{\omega }}_{m} & -u\sigma & -v\sigma \\ -{\bar{\omega }}_{m} & -\frac{\gamma_{m}}{2} & u\rho & v\rho \\ -v\rho & -v\sigma & -(\kappa -2G\cos\theta ) & \Delta +2G\sin\theta \\ u\rho & u\sigma & -(\Delta -2G\sin\theta ) & -(\kappa +2G\cos\theta ) \end{array}\right),$$

where $u=\frac{1}{\sqrt{2}}\left(a_{s}+a_{s}^{*}\right)$, $v=\frac{1}{i\sqrt{2}}\left(a_{s}-a_{s}^{*}\right)$, $\rho =\frac{1}{\sqrt{2}}\left(g^{\prime }+g^{\prime *}\right)$, and $\sigma =\frac{1}{i\sqrt{2}}\left(g^{\prime }-g^{\prime *}\right)$. The eigenvalues of the matrix *A* can be used to determine the dynamic stability of the considered system. In order to stablize the system, the real parts of all the eigenvalues of the matrix *A* must be negative. According to the Routh–Hurwitz stability criterion [26], we find the three stability conditions of the system:

(9) $$\begin{array}{ccc} s_{1} & = & 2\kappa \left(\Delta^{2}-4G^{2}+\kappa^{2}+2\kappa \gamma_{m}\right)+\gamma_{m}\left(\kappa^{2}+2\kappa \gamma_{m}+{\bar{\omega }}_{m}^{2}\right)>0,\\ s_{2} & = & \left(\frac{\gamma_{m}^{2}}{4}+{\bar{\omega }}_{m}^{2}\right)\left(\Delta^{2}-4G^{2}+\kappa^{2}\right)-\left[u^{2}\left(\Delta +2G\sin\theta \right)+v^{2}\left(\Delta -2G\sin\theta \right)-4uvG\cos\theta \right]\\ & & \times \left(\sigma^{2}+\rho^{2}\right){\bar{\omega }}_{m}>0,\\ s_{3} & = & s_{1}\left[\gamma_{m}\left(\Delta^{2}-4G^{2}+\kappa^{2}\right)+\frac{1}{2}\kappa \gamma_{m}^{2}+2\kappa {\bar{\omega }}_{m}^{2}\right]-{\left(2\kappa +\gamma_{m}\right)}^{2}s_{2}>0. \end{array}$$

In the following numerical simulations, we choose the system parameters to satisfy the above stability conditions.

We assume that the incoming noises $a_{in}$ and $b_{in}$ are Gaussian noises, whose mean values are zeros. The time-evolution equations for the fluctuation operators δa and δb have been linearized. Thus, the resulting state of the system is also a Gaussian state and can be fully described by the 4×4 covariance matrix *V* whose elements are given by

(10) $$\begin{array}{c} V_{ij}=\frac{1}{2}\left[\langle U_{i}\left(t\right)U_{j}\left(t\right)\rangle +\langle U_{j}\left(t\right)U_{i}\left(t\right)\rangle \right]. \end{array}$$

Based on Equation (6), we find the evolution equations for the covariance matrix *V*:

(11) $$\begin{array}{c} \frac{dV}{dt}=AV+VA^{T}+D, \end{array}$$

where the diffusion matrix *D* can be calculated with the aid of the correlation functions of the noise operators $D_{ij}=\frac{1}{2}\left[\langle n_{i}\left(t\right)n_{j}\left(t\right)\rangle +\langle n_{j}\left(t\right)n_{i}\left(t\right)\rangle \right]$ and is given by $D=\mathrm{Diag}[\gamma_{m}\left(n_{th}+\frac{1}{2}\right),\gamma_{m}\left(n_{th}+\frac{1}{2}\right),\kappa ,\kappa ]$. The covariance matrix *V* of the system at the steady state can be obtained by solving the following Lyapunov equation [27]:

(12) $$\begin{array}{c} AV+VA^{T}=-D. \end{array}$$

For the Gaussian continuous-variable bipartite system, we can use the logarithmic negativity $E_{N}$ proposed in Ref. [28] to measure the degree of the stationary entanglement between the LG-cavity field and the rotating end mirror, which is given by

(13) $$\begin{array}{c} E_{N}=\max\left[0,-\ln\left(2\eta^{-}\right)\right], \end{array}$$

where $\eta^{-}\equiv \frac{1}{\sqrt{2}}{\left\{\sum \left(V\right)-{\left[\sum {\left(V\right)}^{2}-4\det V\right]}^{1/2}\right\}}^{1/2}$, with $\sum \left(V\right)=\det V_{c}+\det V_{m}-2\det V_{cm}$ and

(14) $$V=\left(\begin{array}{cc} V_{m} & V_{cm}\\ V_{cm}^{T} & V_{c} \end{array}\right).$$

In Equation (14), the 2×2 matrices $V_{m}$ and $V_{c}$ represent the variances of the rotating end mirror and the LG-cavity field, respectively, and the 2×2 matrix $V_{cm}$ describes the correlation between the rotating end mirror and the LG-cavity field. Only when the logarithmic negativity $E_{N}$ is not equal to zero are the LG-cavity field and the rotating end mirror entangled. For the Gaussian continuous-variable bipartite system, there are some other measures of entanglement, such as the Duan’s inseparabiltiy criterion, which is based on the calculation of the total variance of a pair of Einstein–Podolsky–Rosen-type operators [29].

## 4. The Stationary Entanglement of the LG-Cavity Field with the Rotating End Mirror in the Presence of the Nonlinear CK Interaction and the OPA

In the following, we analyze the impacts of the cavity detuning $\Delta_{0}$, the parametric gain *G* and phase θ of the OPA, the strength $g_{ck}$ of the nonlinear CK interaction, the Gaussian laser power *℘*, the topological charge *l* of the cavity field, the mass *m* of the rotating end mirror, and the ambient temperature *T* on the stationary entanglement of the cavity field with the rotating end mirror.

The parameters we use in the numerical calculations are similar to those in Ref. [11], which studies the entanglement of an intracavity LG field with a rotating end mirror: the wavelength of the incident Gaussian laser is λ=810 nm, the cavity length is L=1 mm, the cavity field decays at a rate of κ=2π×1.5 MHz, the rotating end mirror has the radius R=10 μm, the resonance frequency $\omega_{m}=2\pi \times 10$ MHz, the mechanical quality factor $Q_{m}=2\times 10^{6}$, and the damping rate $\gamma_{m}=2\pi \times 5$ Hz. Thus, the studied system is operating in the resolved-sideband limit $\omega_{m}\gg \kappa$.

**Figure 2:**
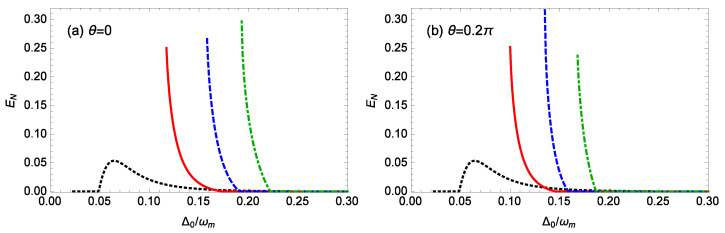
The logarithmic negativity $E_{N}$ against the normalized cavity detuning $\Delta_{0}/\omega_{m}$ for different gains *G* of the OPA when m=100 ng, ℘=1 mW, l=15, $g_{ck}=10^{-3}g$, and T=0.1 K. (**a**) θ=0, (**b**) θ=0.2π. The black-dotted, red-solid, blue-dashed, and green-dot-dashed curves are for G=0, 0.1κ, 0.2κ, 0.3κ, respectively.

Figure 2 plots the logarithmic negativity $E_{N}$ against the normalized cavity detuning $\Delta_{0}/\omega_{m}$ for different gains *G* of the OPA when m=100 ng, ℘=1 mW, θ=0,0.2π, l=15, $g_{ck}=10^{-3}$*g*, and T=0.1 K. When the driving phase θ of the OPA is 0 (Figure 2a), the system stability conditions require $\Delta_{0}/\omega_{m}\geq 0.117,0.158,0.193$ for G=0.1κ, 0.2κ, 0.3κ, respectively, and the corresponding maximum logarithmic negativity $E_{N}$ values just before the unstable regime are 0.250, 0.266, 0.297, respectively. When the driving phase θ of the OPA is set to 0.2π (Figure 2b), the system stability conditions for G=0.1κ, 0.2κ, 0.3κ restrict the normalized cavity detuning to the range of $\Delta_{0}/\omega_{m}\geq 0.100,0.135,0.168$, respectively, the corresponding maximum logarithmic negativity $E_{N}$ just before the unstable regime is 0.252, 0.316, 0.237, respectively. In the absence of the OPA (G=0), the system stability conditions require $\Delta_{0}/\omega_{m}\geq 0.023$, and we observe that the logarithmic negativity $E_{N}$ increases from 0 to a peak value of about 0.05 and then gradually decreases to 0 as the cavity detuning $\Delta_{0}$ increases. Thus, the maximum entanglement in the presence of the OPA far exceeds that in the absence of the OPA (G=0), indicating that the nonlinear gain *G* of the OPA significantly enhances the steady-state entanglement. For a fixed phase θ of the OPA, as the gain *G* of the OPA increases, the regime where the entanglement occurs shifts towards larger cavity detunings $\Delta_{0}$, and the amount of entanglement decreases with increasing cavity detuning $\Delta_{0}$. In Figure 2b, when θ=0.2π, it is noted that the maximum entanglement does not occur at the highest gain *G* of the OPA. This shows the influence of the driving phase θ of the OPA on the entanglement, which is discussed further in the following.

**Figure 3:**
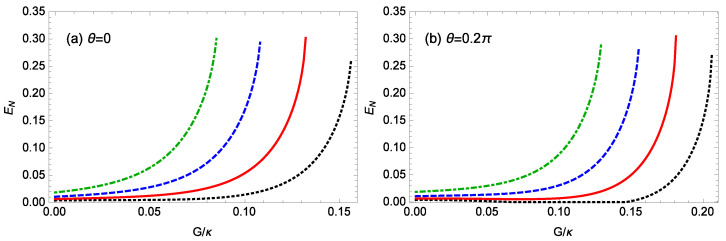
The logarithmic negativity $E_{N}$ against the normalized gain G/κ of the OPA for different strengths $g_{ck}$ of the nonlinear CK interaction when m=100 ng, ℘=1 mW, l=15, $\Delta_{0}=0.12\omega_{m}$, and T=0.1 K. (**a**) θ=0, (**b**) θ=0.2π. The black-dotted, red-solid, blue-dashed, green-dot-dashed curves are for $g_{ck}=0.8\times 10^{-3}g$, $0.9\times 10^{-3}g$, $10^{-3}g$, $1.1\times 10^{-3}g$, respectively.

**Figure 4:**
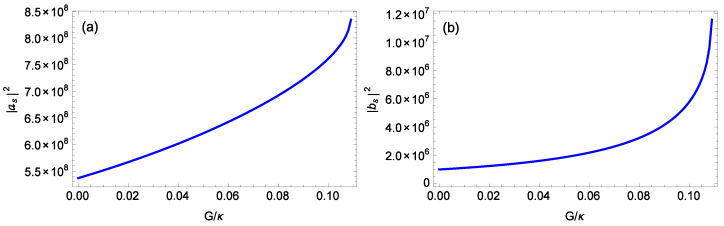
(**a**) The number $|a_{s}{|}^{2}$ of the photons in the cavity field at the steady state against the normalized gain G/κ of the OPA. (**b**) The number $|b_{s}{|}^{2}$ of the phonons in the rotating end mirror at the steady state against the normalized gain G/κ of the OPA. The parameters are m=100 ng, ℘=1 mW, l=15, $\Delta_{0}=0.12\omega_{m}$, $g_{ck}=10^{-3}g$, and θ=0.

Figure 3 plots the logarithmic negativity $E_{N}$ against the normalized gain G/κ of the OPA for different strengths $g_{ck}$ of the nonlinear CK interaction when m=100 ng, ℘=1 mW, θ=0,0.2π, l=15, $\Delta_{0}=0.12\omega_{m}$, and T=0.1 K. When θ=0 (Figure 3a), for $g_{ck}=0.8\times 10^{-3}g$, $0.9\times 10^{-3}g$, $10^{-3}g$, $1.1\times 10^{-3}g$, when the gain *G* of the OPA satisfies G/κ≤ 0.156, 0.132, 0.108, 0.085, respectively, the system remains stable. For $g_{ck}=0.8\times 10^{-3}g$, $0.9\times 10^{-3}g$, $10^{-3}g$, $1.1\times 10^{-3}g$, as the gain *G* of the OPA increases, the logarithmic negativity $E_{N}$ increases and reaches the maximum values of about 0.263, 0.302, 0.293, and 0.300, respectively, just before entering the unstable regime. In Figure 3b (θ=0.2π), for $g_{ck}/g=0.8\times 10^{-3}$, $0.9\times 10^{-3}$, $1.0\times 10^{-3}$, $1.1\times 10^{-3}$, the stability condition imposes the limitation G/κ≤ 0.206, 0.181, 0.155, 0.129, respectively. The maximum logarithmic negativity $E_{N}$ values before approaching the unstable regime are about 0.273, 0.305, 0.279, and 0.292, respectively. This demonstrates that the gain *G* of the OPA can effectively enhance the steady-state entanglement between the cavity field and the rotating end mirror. This is due to the fact that increasing the gain *G* of the OPA leads to a larger photon number $|a_{s}{|}^{2}$ in the cavity field and a larger phonon number $|b_{s}{|}^{2}$ in the rotating end mirror, as shown in Figure 4, which plots the number $|a_{s}{|}^{2}$ of the photons in the cavity field and the number $|b_{s}{|}^{2}$ of the phonons in the rotating end mirror at the steady state against the normalized gain G/κ of the OPA when m=100 ng, ℘=1 mW, θ=0, l=15, $\Delta_{0}=0.12\omega_{m}$, and $g_{ck}=10^{-3}g$. A larger photon number $|a_{s}{|}^{2}$ in the cavity field increases the coupling between the cavity field and the rotating end mirror. Meanwhile, a larger photon number $|a_{s}{|}^{2}$ in the cavity field and a larger phonon number $|b_{s}{|}^{2}$ in the rotating end mirror give rise to a stronger CK coupling between the cavity field and the rotating end mirror. Moreover, in Figure 3, for fixed values of θ and *G*, the larger the CK coupling strength $g_{ck}$, the larger the entanglement, but, for a fixed value of θ, with increasing the CK coupling strength $g_{ck}$, the stable regime becomes narrower. Comparing Figure 3a with Figure 3b, it is evident that changing the driving phase θ of the OPA significantly alters the regime of steady-state entanglement.

**Figure 5:**
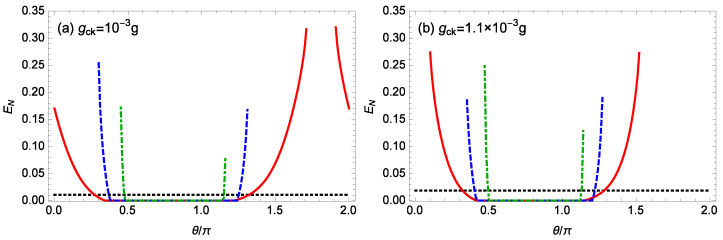
The logarithmic negativity $E_{N}$ against the phase θ/π of the OPA for different gains *G* of the OPA when m=100 ng, ℘=1 mW, l=15, $\Delta_{0}=0.12\omega_{m}$, and T=0.1 K. (**a**) $g_{ck}=10^{-3}g$, (**b**) $g_{ck}=1.1\times 10^{-3}g$, The black-dotted, red-solid, blue-dashed, green-dot-dashed curves are for G=0,0.1κ,0.2κ,0.3κ, respectively.

Figure 5 plots the logarithmic negativity $E_{N}$ against the phase θ/π of the OPA for some different gains *G* of the OPA when m=100 ng, ℘=1 mW, l=15, $\Delta_{0}=0.12\omega_{m}$, $g_{ck}=10^{-3}g,1.1\times 10^{-3}g$, and T=0.1 K. Without the OPA (G=0), for $g_{ck}=10^{-3}g,1.1\times 10^{-3}g$, when the phase θ of the OPA is changed from 0 to 2π, the values of the logarithmic negativity $E_{N}$ remain unchanged, which are about 0.011, 0.019, respectively. Next we look at the case with the OPA. When $g_{ck}=10^{-3}g$ (Figure 5a), for G=0.1κ,0.2κ,0.3κ, the system is stable when the phase θ of the OPA satisfies θ∈[0,1.71π]⋃[1.91π,2π], θ∈[0.3π,1.31π], and θ∈[0.45π,1.16π], respectively. When $g_{ck}=1.1\times 10^{-3}g$ (Figure 5b), for G=0.1κ,0.2κ,0.3κ, the system is stable when the phase θ of the OPA satisfies θ∈[0.1π,1.52π], θ∈[0.35π,1.27π], and θ∈[0.47π,1.14π], respectively. Thus, for a fixed strength $g_{ck}$ of the nonlinear CK interaction, increasing the gain *G* of the OPA makes the system stable in a narrower range of phases θ. And when the gain *G* of the OPA is fixed, for $g_{ck}=1.1\times 10^{-3}g$, the system is stable in a narrower range of phases θ compared to that for $g_{ck}=10^{-3}g$. When $g_{ck}=10^{-3}g$ (Figure 5a), for G=0.1κ,0.2κ,0.3κ, the logarithmic negativity $E_{N}$ takes its maximum values of about 0.320, 0.254, and 0.171 at θ=1.91π,0.3π,0.45π, respectively. When $g_{ck}=1.1\times 10^{-3}g$ (Figure 5b), for G=0.1κ,0.2κ,0.3κ, the logarithmic negativity $E_{N}$ takes its maximum value of about 0.274, 0.190, and 0.249 at θ=0.1π,1.27π,0.47π, respectively. Hence, for a fixed value of the CK coupling strength $g_{ck}$, it is noted that the presence of the OPA can enhance the entanglement since the logarithmic negativity $E_{N}$ in the presence of the OPA can be larger than that in the absence of the OPA, but the maximum entanglement does not increase with an increasing gain *G* of the OPA, and the maximum entanglement happens at a phase θ of the OPA at which the system is close to the unstable regime.

**Figure 6:**
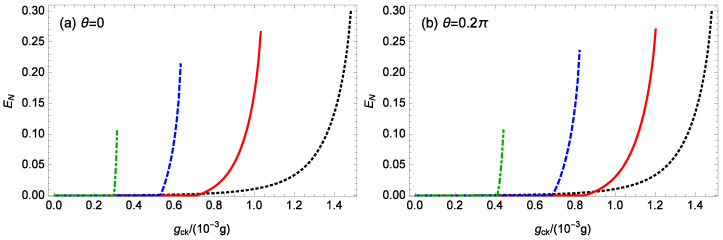
The logarithmic negativity $E_{N}$ against the normalized strength $g_{ck}/\left(10^{-3}g\right)$ of the nonlinear CK interaction for different gains *G* of the OPA when m=100 ng, ℘=1 mW, l=15, $\Delta_{0}=0.12\omega_{m}$, and T=0.1 K. (**a**) θ=0, (**b**) θ=0.2π. The black-dotted, red-solid, blue-dashed, and green-dot-dashed curves are for G=0, 0.1κ, 0.2κ, 0.3κ, respectively.

Figure 6 depicts the logarithmic negativity $E_{N}$ versus the normalized strength $g_{ck}/\left(10^{-3}g\right)$ of the nonlinear CK interaction for different gains *G* of the OPA when m=100 ng, θ=0, 0.2π, ℘=1 mW, l=15, $\Delta_{0}=0.12\omega_{m}$, and T=0.1 K. When θ=0 (Figure 6a), for different OPA nonlinear gains G=0,0.1κ,0.2κ,0.3κ, the system stability requires $g_{ck}/\left(10^{-3}g\right)\leq$1.48, 1.03, 0.63, 0.312. As the strength $g_{ck}$ of the nonlinear CK interaction increases, the logarithmic negativity $E_{N}$ for different *G* values rapidly rises from 0, reaching a maximum value of about 0.299, 0.265, 0.213, 0.104, respectively, just before the system approaches instability. Similarly, in Figure 6b (θ=0.2π), for G=0.1κ, 0.2κ, 0.3κ, the system stability conditions require $g_{ck}/\left(10^{-3}g\right)\leq 1.48$, 1.20, 0.82, 0.44, respectively. As the strength $g_{ck}$ of the nonlinear CK interaction increases, the maximum logarithmic negativity $E_{N}$ values before approaching instability are about 0.299, 0.270, 0.235, 0.106, respectively. Thus, for a fixed phase θ of the OPA, with an increasing gain *G* of the OPA, the instability is triggered at a weaker strength $g_{ck}$ of the nonlinear CK interaction. For a given nonzero gain *G* of the OPA, it is noted that the instability for θ=0.2π is triggered at a stronger strength $g_{ck}$ of the nonlinear CK interaction compared to the case for θ=0. For a fixed phase θ and gain *G* of the OPA, it is possible to improve the entanglement by increasing the strength $g_{ck}$ of the nonlinear CK interaction. Additionally, as the gain *G* of the OPA increases, the entanglement appears at a weaker strength $g_{ck}$ of the nonlinear CK interaction and reaches the maximum value at a weaker strength $g_{ck}$ of the nonlinear CK interaction, but the maximum entanglement decreases.

**Figure 7:**
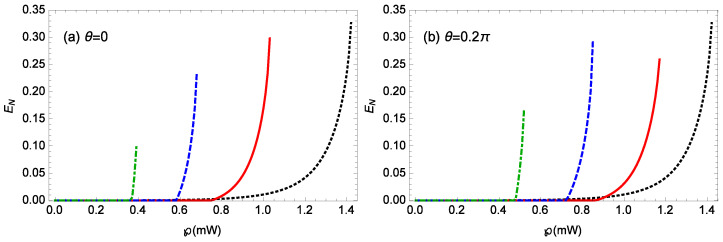
The logarithmic negativity $E_{N}$ against the laser power *℘* for different gains *G* of the OPA when m=100 ng, $\Delta_{0}=0.12\omega_{m}$, $g_{ck}=10^{-3}g$, l=15, and T=0.1 K. (**a**) θ=0, (**b**) θ=0.2π. The black-dotted, red-solid, blue-dashed, green-dot-dashed curves are for G=0,0.1κ,0.2κ,0.3κ, respectively.

Figure 7 plots the logarithmic negativity $E_{N}$ against the laser power *℘* for different gains *G* of the OPA when m=100 ng, θ=0,0.2π, $\Delta_{0}=0.12\omega_{m}$, $g_{ck}=10^{-3}g$, l=15, and T=0.1 K. When θ=0 (Figure 7a), for G=0, 0.1κ, 0.2κ, 0.3κ, the stability conditions require ℘≤ 1.42, 1.03, 0.68, 0.39 mW, and the corresponding driving power thresholds for generating entanglement are 0.36, 0.76, 0.59, 0.37 mW, the maximum logarithmic negativity $E_{N}$ values near the unstable regime are 0.326, 0.298, 0.236, and 0.098, respectively. Similarly, when θ=0.2π (Figure 7b), the stability conditions require ℘≤ 1.42, 1.17, 0.85, 0.52 mW, respectively, and the corresponding driving power thresholds for generating entanglement are 0.36, 0.87, 0.73, 0.48 mW, respectively; then, the maximum logarithmic negativity $E_{N}$ values near the unstable regime are about 0.326, 0.259, 0.293, 0.168, respectively. For a fixed phase θ of the OPA, from Equation (4), it is noted that increasing the laser power *℘* can enhance the photon number $|a_{s}{|}^{2}$ within the cavity; thus, it is possible to improve the steady-state entanglement by increasing the laser power *℘*. Additionally, as the gain *G* of the OPA increases, the entire entanglement regime shifts towards lower laser powers, with the threshold power appearing at lower values. This indicates that it is possible to achieve stronger steady-state entanglement with lower laser powers.

**Figure 8:**
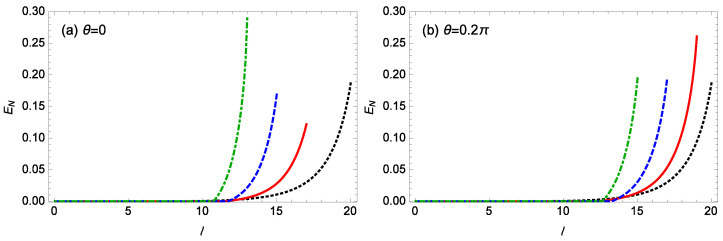
The logarithmic negativity $E_{N}$ against the topological charge *l* of the cavity field for different gains *G* of the OPA when m=100 ng, ℘=1 mW, $\Delta_{0}/\omega_{m}=0.12$, $g_{ck}=10^{-3}g$, and T=0.1 K. (**a**) θ=0, (**b**) θ=0.2π. The black-dotted, red-solid, blue-dashed, green-dot-dashed curves are for G=0,0.05κ,0.1κ,0.15κ, respectively.

Figure 8 plots the logarithmic negativity $E_{N}$ against the topological charge *l* of the cavity field for different gains *G* of the OPA when m=100 ng, θ=0,0.2π, ℘=1 mW, $\Delta_{0}/\omega_{m}=0.12$, $g_{ck}=10^{-3}g$, and T=0.1 K. When θ=0 (Figure 8a), for G=0,0.05κ,0.1κ,0.15κ, the entanglement occurs only when the topological charge *l* is not less than the threshold values $l_{c}=8$, 12, 12, 11, respectively, the stable regime is found to be l≤ 20, 17, 15, 13, and the maximum logarithmic negativity $E_{N}$ close to the unstable regime is about 0.189, 0.122, 0.170, 0.292, respectively. When θ=0.2π (Figure 8b), for G=0, 0.05κ, 0.1κ, 0.15κ, the entanglement appears only when the topological charge *l* is not less than the threshold value $l_{c}=8$, 13, 14, 13, respectively, and the stable regime is found to be l≤20, 19, 17, 15, respectively, the maximum logarithmic negativity $E_{N}$ close to the unstable regime is about 0.188, 0.261, 0.195, 0.196, respectively. Notably, when the topological charge *l* is less than the threshold value $l_{c}$, the optorotational coupling is not strong enough to generate the entanglement. In addition, the exchange of orbital angular momentum between photons and helical phase elements is fundamental for generating entanglement between the cavity and mechanical modes. Thus, it is possible that an increase in the topological charge *l* facilitates the enhancement of entanglement. It is found that a higher gain *G* of the OPA can result in the maximum entanglement occurring at a smaller topological charge *l*, with a more rapid increase in entanglement and a narrowing stable regime. For a fixed nonzero gain *G* of the OPA, the system for θ=0.2π becomes unstable at a larger topological charge *l* compared to the case for θ=0.

**Figure 9:**
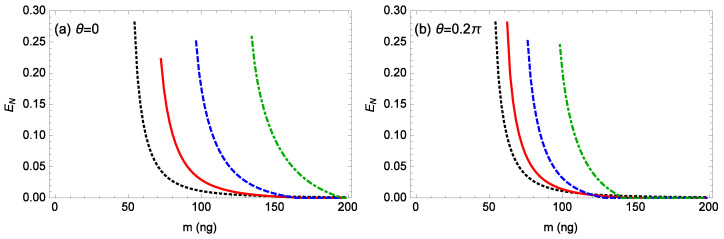
The logarithmic negativity $E_{N}$ against the mass *m* of the rotating end mirror for different gains *G* of the OPA when ℘=1 mW, $\Delta_{0}/\omega_{m}=0.12$, $g_{ck}=10^{-3}g$, l=15, and T=0.1 K. (**a**) θ=0, (**b**) θ=0.2π. The black-dotted, red-solid, blue-dashed, green-dot-dashed curves are for G=0,0.05κ,0.1κ,0.15κ, respectively.

Figure 9 shows the logarithmic negativity $E_{N}$ against the mass *m* of the rotating end mirror for different gains *G* of the OPA when ℘=1 mW, θ=0,0.2π, $\Delta_{0}/\omega_{m}=0.12$, $g_{ck}=10^{-3}g$, l=15, and T=0.1 K. When θ=0 (Figure 9a), for G=0, 0.05κ, 0.1κ, 0.15κ, the system’s stability conditions require m≥54, 72, 96, 134 ng, respectively, and the logarithmic negativity $E_{N}$ reaches the maximum values of about 0.281, 0.222, 0.251, 0.258 near the unstable regime, respectively. When θ=0.2π (Figure 9b), for G=0, 0.05κ, 0.1κ, 0.15κ, the system’s stability conditions require m≥54, 62, 76, 98 ng, respectively, and the logarithmic negativity $E_{N}$ reaches the maximum values of about 0.281, 0.281, 0.251, 0.245 near the unstable regime, respectively. For fixed values of θ and *G*, when the mass *m* of the rotating end mirror increases, the entanglement decreases. When the phase θ of the OPA is fixed, for a larger gain *G* of the OPA, the system becomes stable at a larger mass *m* of the rotating end mirror. For a fixed nonzero gain *G* of the OPA, the system for θ=0.2π becomes stable at a smaller mass *m* of the rotating end mirror in comparison with the case for θ=0. When the phase θ of the OPA is fixed, a larger gain *G* of the OPA can generate the entanglement between the optical mode and a rotating end mirror with a larger mass *m*. Even at around 150 ng (Figure 9a), the entanglement can still be achieved, providing valuable insights for experimental realization.

**Figure 10:**
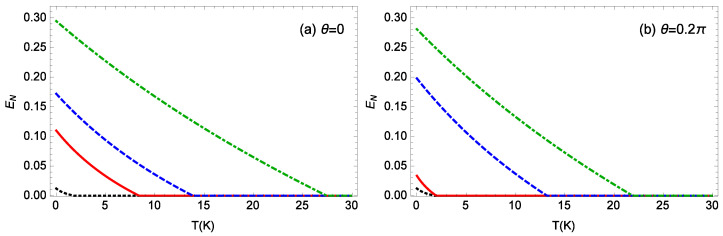
The logarithmic negativity $E_{N}$ against the ambient temperature *T* for different gains *G* of the OPA when m=100 ng, ℘=1 mW, l=15, $\Delta_{0}/\omega_{m}=0.12$, and $g_{ck}=10^{-3}g$. (**a**) θ=0, (**b**) θ=0.2π. The black-dotted, red-solid, blue-dashed, green-dot-dashed curves in (**a**) are for G=0,0.09κ,0.1κ,0.108κ, respectively. The black-dotted, red-solid, blue-dashed, green-dot-dashed curves in (**b**) are for G=0,0.1κ,0.15κ,0.155κ, respectively.

Figure 10 plots the logarithmic negativity $E_{N}$ against the ambient temperature *T* for different gains *G* of the OPA when m=100 ng, θ=0,0.2π, ℘=1 mW, l=15, $\Delta_{0}/\omega_{m}=0.12$, and $g_{ck}=10^{-3}g$. For θ=0,0.2π, the system is stable when *G* is less than 0.109κ, 0.156κ, respectively. Thus, the system for θ=0.2π becomes unstable at a larger gain *G* of the OPA in contrast to the case for θ=0. When θ=0 (Figure 10a), for G=0,0.09κ,0.1κ,0.108κ, the logarithmic negativity $E_{N}$ takes its maximum values of about 0.012, 0.110, 0.172, and 0.295 at T=0 K, respectively, and the logarithmic negativity $E_{N}$ is zero when the ambient temperature *T* is not less than 1.8 K, 8.3 K, 13.8 K, and 27.4 K, respectively. When θ=0.2π (Figure 10b), for G=0,0.1κ,0.15κ,0.155κ, the logarithmic negativity $E_{N}$ takes its maximum value of about 0.012, 0.034, 0.198, and 0.281 at T=0 K, respectively, and the logarithmic negativity $E_{N}$ is zero when the ambient temperature *T* is not less than 1.8 K, 1.9 K, 13.1 K, and 21.8 K, respectively. When the phase θ and gain *G* of the OPA are fixed, as the ambient temperature *T* increases, the entanglement decreases and eventually vanishes, which can be attributed to the detrimental effect of the thermal noise of the environment on the entanglement. For a fixed phase θ of the OPA, an increase in the gain *G* of the OPA results in a larger entanglement and allows the entanglement to persist at higher temperatures. This indicates that the gain *G* of the OPA not only enhances the steady-state entanglement, but also improves the system’s ability to withstand decoherence in a thermal environment.

In our previous work [20], it has been shown that the entanglement between the cavity field and the rotating end mirror with mass 100 ng can be improved by using the nonlinear CK interaction, and the entanglement $\left(E_{N}=0.459\right)$ can be obtained with the nonlinear CK interaction $\left(g_{ck}=10^{-3}g\right)$, which is much larger than the entanglement $\left(E_{N}=0.011\right)$ (Figure 5a) between the cavity field and the rotating end mirror with mass 100 ng in the presence of the nonlinear CK interaction $\left(g_{ck}=10^{-3}g\right)$ but in the absence of the OPA in this work. The reason is that a large topological charge l=70 of the cavity field is used in Ref. [20] and a small topological charge l=15 of the cavity field are used in this work. Furthermore, it has been shown in this work that a larger entanglement $\left(E_{N}=0.320\right)$ (Figure 5a) between the cavity field and the rotating end mirror can be obtained under the combined action of the nonlinear CK interaction $\left(g_{ck}=10^{-3}g\right)$ and the OPA (G=0.1κ,θ=1.91π).

Finally, we discuss the possibility of the experimental feasibility of this proposal. The optorotational entanglement can be verified by measuring the logarithmic negativity $E_{N}$, which requires one to measure the 10 independent elements of the correlation matrix *V* [27]. The quadratures of the LG-cavity mode can be measured by homodyne detecting the cavity output field [27]. And the quadratures of the rotating end mirror can be measured by homodyning the output field from the adjacent cavity formed by the rotating end mirror and a third fixed spiral phase element [27]. On the other hand, the LG-cavity optorotational system has not been realized experimentally yet [12]. However, it has been demonstrated experimentally that an LG laser beam with a topological charge of l=100 can be achieved by using spiral-phase mirrors [30], that the torsional frequency of a nanomechanical resonator can be up to 8.4 MHz [31], and that a micromechanical resonator with mass 25 ng, radius 15 μm, and mechanical quality factor $1.3\times 10^{5}$ can be cooled to 135 mK [32]. Thus, with the rapid development of fabrication technologies, it is possible to experimentally realize the optorotational entanglement in the LG-cavity optorotational system with the rotating mirror having mass 100 ng, radius 10 μm, frequency 2π×10 MHz, and mechanical quality factor $2\times 10^{6}$ at temperature T=0.1 K.

## 5. Conclusions

To sum up, we have investigated the stationary entanglement between an LG-cavity field and a rotating end mirror in an LG-cavity optorotational system with a nonlinear CK interaction and a degenerate OPA. We show that the combination of the nonlinear CK interaction and the OPA have detrimental effects on the system stability so that the system becomes unstable at a lower Gaussian laser power, a smaller topological charge of the cavity field, and a larger mass of the rotating end mirror. Moreover, we show that the strength of the nonlinear CK interaction and the gain and phase of the OPA have strong impacts on the stationary entanglement. We find that the combination of the nonlinear CK interaction and the OPA can significantly increase the entanglement at lower Gaussian laser powers, the smaller topological charges of the cavity field, and the larger masses of the rotating end mirror and make the entanglement more robust against the ambient temperature compared to the case in the presence of only the nonlinear CK interaction [20]. And the maximum entanglement appears near the unstable regime in the presence of the nonlinear CK interaction and the OPA. In contrast, the injection of two-level atoms into the optical cavity of an LG-cavity optorotational system can also significantly improve the optorotational entanglement and enhance the robustness of entanglement to the ambient temperature, but the maximum entanglement does not happen near the unstable regime in the presence of two-level atoms [12]. Our findings have important implications for realizing the stationary entanglement experimentally. In the near future, we might study how the combination of the nonlinear CK interaction and the OPA affects the entanglement of multiple rotating mirrors in an LG-cavity optorotational system [33].

## Data Availability

Data are contained within the article.
